# Where Shall the Line Be Drawn?

**Published:** 1892-08

**Authors:** 


### Editorial.

### WHERE SHALL THE LINE BE DRAWN?

A QUESTION of some importance, viewed from a professional standpoint, is that of the admission of proprietary advertisements to the pages of dental and medical journals.

It is a recognized axiom of medical ethics that it is derogatory to professional character for a physician to hold a patent for any surgical instrument or medicine, or to dispense a *secret nostrum*, whether it be the composition or exclusive property of himself or of others. Carry this law to its legitimate conclusion, and it must be equally a violation of the code for medical journals to receive and print advertisements of the character condemned. Yet there is probably not a medical or dental journal published that does not admit advertisements open to criticism.

We quote abstracts upon another page from a paper read by Dr. S. Solis-Cohen before the Philadelphia County Medical Society, and also portions of the able rejoinder from the pen of the editor of the *Journal of the American Medical Association.*

That part of this answer devoted to the financial side of the question, while forcibly stated, is necessarily weak, in that it defends the practice on the ground of necessity. The retort may safely be made, "If journals cannot be supported free from the taint of connivance with empiricism, then better no journals," and it is presumed that if this course were adopted the majority of professional periodicals would cease to exist.

The question naturally follows, If advertisements be necessary to the life of a journal, can they be so prepared that they will not impinge upon any code of ethics, dental or medical?

In considering this subject in its entirety, it is obvious that it should not be met by a narrow interpretation of the code on the one hand, or a too liberal rendering on the other. The question of finance should, it seems to us, be left out altogether. However important this may be, we are not prepared to believe that a professional journal may not be carried on without the aid of questionable pecuniary methods. It is recognized that before this can be accomplished an entire change will be necessary in professional sentiment, but to assert its impossibility is to acknowledge an entire absence of professional spirit. The latter exists more pronounced, we judge, in medical than in dental circles, yet, in degree, latent in both, and may need a professional convulsion to bring it into activity. The indifference manifested in the financial success of journals devoted to professional work is well known, and requires no extended illustration. It seems to be taken for granted that they are not subject to the ordinary laws of business, but have a life superior to the traffic in dollars. It is the business of every man connected with a profession to be interested in the journals published for its benefit, and to know something of the efforts made to meet the current demands against it. If each were to assume a share of this financial responsibility, it would not be long before these periodicals would be above the necessity of depending upon outside help for their existence. Until this is accomplished the keen edge of criticism could be turned with advantage towards the profession.

Admitting that medical and dental journalism has not reached its millenial stage, and must, for a long time in the future, accept the patronage of those who deal in supplies, is it not proper to consider where the line may be drawn, so that the journal may not be lowered in its professional standard.

The fact does not seem to be recognized with sufficient force, that methods of manufacture have been so improved in the past half-century, and especially in the last decade, that remedies are placed to-day in the hands of the practitioner in a form and with a degree of perfection not heretofore possible of attainment. It requires no argument to prove that private hands are not equal to the perfection of combination accomplished by capital.

When Dr. S. Solis-Cohen classes an entire series of valuable preparations with the recognized empiric remedies, it seems to us he does an injustice not only to the manufacturer, but to the medical practitioner. To our comprehension the active agent placed on the market in a form pleasant to patient and satisfactory to the dispenser, and with the stamp of a responsible firm as proof of chemical purity, is preferable to adulteration made possible through the ordinary channels.

The medical side of the question does not interest us as much as that of the dental, but both are so intermingled that it is difficult, if not impossible, to separate them.

Dentistry has from its earliest beginning been subject to the overpowering influence of trade. The amalgams, plastics, and even the gold used, has been prepared for us by processes unknown. To this day no one, as far as we are aware, has given the process of preparing true non-cohesive gold. When amalgams were first introduced, each individual manufactured his own, and the result was that they were denounced and men read out of the profession for using them. Not until the manufacture was conducted on a large scale, and attention paid to chemical purity of metals, could these be used as now to the mutual advantage of patient and operator. The same is true of oxy-phosphate and oxy-chloride of zinc, and also of gutta-percha. The processes of preparation of these are secret, and their manufacture holds the same relation to dentistry as those mentioned by Dr. Solis-Cohen do to medicine.

To our way of thinking, responsible houses in both medicine and dentistry have performed a most valuable service in producing preparations impossible of accomplisment by the individual. While this is stated, we recognize the fact that these houses are in duty bound to give to the professions the agents used in the combinations. If this is not done, they must be relegated to that other class with whom no respectable journal can fraternize.

The individual who brings together, in a secret formula, a series of well-known ingredients and advertises the result as a specific, is an empiric of formidable proportions. They present themselves prominently as "obtunders of sensitive dentine for extraction locally without pain," and in washes and tooth-powders. Between these and the first-named, it seems to us, a line, and a very wide one, should be drawn. The latter should have no place in a well-regulated journal, while the former should not only be there, but, as is clearly stated by the editor of the organ of the American Medical Association, practitioners "can by their use present their patrons much more elegant specimens of pharmaceutical work," and we can add dental preparations.

What is needed in considering this question is to understand that we are in the beginning, apparently, of a period when the individual is to be submerged in the body of workers, and that this will apply in the near future to all the relations of professional and business life. To fight against this tendency seems an unprofitable contention, if indeed it may not be a direct blow to professional progress.

